# Barriers and Enablers to Engaging with Long-Term Follow-Up Care Among Canadian Survivors of Pediatric Cancer: A COM-B Analysis

**DOI:** 10.3390/curroncol32080427

**Published:** 2025-07-30

**Authors:** Holly Wright, Sharon H. J. Hou, Brianna Henry, Rachelle Drummond, Kyle Mendonça, Caitlin Forbes, Iqra Rahamatullah, Jenny Duong, Craig Erker, Michael S. Taccone, R. Liam Sutherland, Paul C. Nathan, Maria Spavor, Karen Goddard, Kathleen Reynolds, Sharon Paulse, Annette Flanders, Fiona S. M. Schulte

**Affiliations:** 1 Department of Oncology, Cumming School of Medicine, University of Calgary, Calgary, AB T2N 4N1, Canada; 2 Faculty of Medicine, University of Alberta, Edmonton, AB T6G 2R3, Canada; 3 BC Children’s Hospital, Vancouver, BC V6H 3N1, Canada; 4 Faculty of Education, Simon Fraser University, Burnaby, BC V5A 1S6, Canada; 5 Alberta Children’s Hospital, Calgary, AB T3B 6A8, Canada; 6 Department of Pediatrics, Dalhousie University, Halifax, NS B3H 4R2, Canada; 7 Childhood Cancer Survivor Canada, Toronto, ON M5H 3R3, Canada; 8 Division of Neurosurgery, Department of Surgery, Faculty of Medicine, University of Ottawa, Ottawa, ON K1N 6N5, Canada; 9 Department of Community Health Sciences, Cumming School of Medicine, University of Calgary, Calgary, AB T2N 2T8, Canada; 10 Department of Pediatrics, Institute for Health Policy, Management and Evaluation, University of Toronto, Toronto, ON M5S 1A1, Canada; 11 Department of Pediatrics, Faculty of Medicine and Dentistry, University of Alberta, Edmonton, AB T6G 2R3, Canada; 12 Department of Surgery, Faculty of Medicine, The University of British Columbia, Vancouver, BC V6T 1Z4, Canada; 13 Department of Family Medicine, Cumming School of Medicine, University of Calgary, Calgary, AB T2N 2T8, Canada; 14 Long Term Survivors Clinic, Alberta Children’s Hospital, Calgary, AB T2N 4N1, Canada; 15 BC Cancer, Vancouver, BC V5Z 4J7, Canada; 16 Long Term Follow Up Clinic, IWK Health, Halifax, NS B3K 6R8, Canada

**Keywords:** barriers, enablers, cancer survivorship, pediatric oncology, long-term follow up, COM-B model

## Abstract

Survivors of pediatric cancer are at risk for late effects and require risk-adapted long-term follow-up (LTFU) care. Yet less than 50% of survivors attend LTFU care. This study aimed to identify barriers and enablers of engaging with LTFU care as perceived by Canadian survivors of pediatric cancer and healthcare providers (HCPs). Survivors (*n* = 108) and HCPs (*n* = 20) completed surveys assessing barriers and enablers to attending LTFU care, summarized using descriptive statistics. Participants were invited to participate in survivor focus groups (*n* = 22) or HCP semi-structured interviews (*n* = 7). These were analyzed using reflexive thematic analysis and the Capability, Opportunity, and Motivation for Behaviour Change (COM-B) model, which explores how an individual’s *capability*, *opportunity*, and *motivation* influence a target behaviour. Structural barriers, transitioning from pediatric to adult care, and time constraints were highlighted as barriers that affect survivors’ *physical opportunity* to engage in LTFU care. Accessibility, financial support, HCPs and family support, and community resources were highlighted as enablers that better survivors’ *physical* and *social opportunity* to engage in LTFU care. In conclusion, Canadian survivors of pediatric cancer highlighted barriers that limited their *physical opportunity* to attend LTFU care, while factors that enhanced their *physical* and *social opportunities* facilitated greater engagement with LTFU care.

## Simple Summary

Survivors of pediatric cancer are at risk for late effects and require risk-adapted long-term follow-up (LTFU) care. Yet less than 50% of survivors attend LTFU care, highlighting the need to understand the barriers and enablers they face. While barriers and enablers to LTFU engagement have been explored internationally, there is a lack of research capturing the unique experiences of Canadian survivors, given Canada’s distinct geographical and publicly funded healthcare context. This study found that structural barriers, transitioning from pediatric to adult care, and time constraints were themes highlighted as barriers that prevent survivors’ engaging with LTFU care, while increasing accessibility, financial support, supportive relationships with healthcare providers and family, and community support were themes highlighted as enablers to engaging in LTFU care. Future interventions targeting the identified barriers and enablers have the potential to improve survivors’ ability to attend LTFU care, allowing for improved surveillance and treatment of late effects.

## 1. Introduction

Every year in Canada, approximately 1000 children under the age of 15 are diagnosed with cancer, with 84% surviving at least five years post-diagnosis [1]. As a result of their cancer and its treatment, survivors are at an increased risk of long-term physical (e.g., cardiomyopathies, cataracts, infertility) and psychological (e.g., neurocognitive delay, post-traumatic stress) late effects [2,3,4]. It is crucial that survivors of pediatric cancer receive individualized and cancer-specific long-term follow-up (LTFU) care after their diagnosis and treatment to survey for and treat any late effects that may occur. However, 46% of long-term survivors of pediatric cancer do not receive the cancer-specific follow-up care they need, suggesting that there are potential barriers to LTFU care attendance [5]. These challenges may be perpetuated among young adult survivors of pediatric cancer (18–39 years old) as they face many life changes in this developmental period, including identity development, educational and vocational attainment, and interpersonal relationships [6]. While navigating this time of change, young adult survivors are also increasingly given more responsibility in terms of their healthcare [7]. These factors that young adult survivors face may limit their engagement with LTFU care [7]. It is crucial that we understand what may be limiting (barriers) and what may be helping (enablers) young adult survivors attend LTFU care to increase their engagement.

Previously identified barriers to attending LTFU care for adult survivors of pediatric cancer included a lack of information about potential late effects and the importance of LTFU care [7,8,9,10,11,12,13,14,15,16], structural barriers such as travel time and distance to the clinic [3,7,8,9,17,18,19], time constraints [3,9,16,17,18], financial barriers such as cost and insurance [7,11,12,13,14,16,18,20,21], lack of communication from or mistrust in their healthcare team [3,19,22], and anxiety with attending appointments [7,8,9,13,18,19]. Enablers to accessing LTFU care for adult survivors of pediatric cancer include fear of another cancer diagnosis [10], knowledge about the importance of LTFU care [7], belief in the importance of LTFU care [7,10,19], reminders [10,19], access to specialized care [9], a familiar healthcare team [19], expressing gratitude to physicians [9], and reassurance about their current health status [9].

Healthcare providers (HCPs) are an integral part of providing LTFU care and it is crucial that they understand the current or potential barriers and enablers to attending LTFU care that their patients face. There is an awareness from HCPs that survivors face barriers to accessing LTFU care [19,23,24,25,26,27]. HCPs identified travel time or distance from the clinic, insurance coverage, psychological barriers or resistance to LTFU care, privacy issues, and lack of knowledge about late effects and the importance of LTFU care as perceived barriers [25,26,28]. However, there is a lack of literature identifying HCPs’ perception of perceived enablers.

Importantly, existing research focusing on barriers and enablers from a survivor’s point of view has largely resulted from the United States, with no Canadian literature identifying national barriers and enablers of attendance [3,11,12,18,19,21,29]. While many findings may be applicable to the Canadian context, Canadian survivors face a unique set of barriers and enablers due to the geography and national healthcare system of Canada. For instance, cost of treatment (e.g., insurance cost) is often cited as a significant barrier in the literature in the United States; however, Canada has federal universal healthcare coverage, which vastly influences the way survivors interact with LTFU [7,11,12,13,14,16,18,20,21]. In Canada, the organization of healthcare systems, including LTFU care, is at the provincial level [30,31]. Some provinces provide LTFU to survivors via family physicians, while other provinces, such as Alberta, provide LTFU via specialist-led (often pediatric oncologist) hospital LTFU clinics [30,31]. Canada’s healthcare system presents distinct barriers, including prolonged wait times that cannot often be circumvented within the public sector, and challenges in coordinating care across provincial jurisdictions [32]. As such, survivors experience differing care based on location in Canada, highlighting a variety of experiences whose voices should be captured. Furthermore, the vast geographical landscape of Canada results in many survivors who may travel long distances (>100 km) to reach care, often from places with limited or no public transport [33]. For example, the Northwest Territories do not have a LTFU clinic in the province and requires survivors to travel to the Stollery Children’s Hospital in Alberta for care [30]. These residents often may need government-funded transport, including air or land travel, to reach medically necessary care [34]. Further research on how these factors interact to affect Canadian survivors’ ability to attend LTFU care is warranted.

Furthermore, there has been limited use of conceptual frameworks to help guide a deeper understanding of survivors’ access to LTFU care [10]. The Capability, Opportunity and Motivation for Behaviour Change (COM-B) model works to understand how an individual’s capability, opportunity, and motivation influence a target behaviour [35]. *Capability* is defined as an individual’s physical (e.g., skills) and psychological (e.g., memory) capability to engage with a behaviour [35]. *Opportunity* refers to the characteristics of an individual’s physical or social environment that facilitate a behaviour [35]. *Motivation* refers to the reflective (e.g., planning) and autonomic (e.g., emotions) brain processes that energize behaviour [35]. This model recognizes how behaviour can be influenced by any combination of the three factors, allowing for more targeted intervention to address behaviour change [35]. The extant literature has used the COM-B model to understand how barriers and enablers have affected different behaviours in the adult cancer population to provide a basis for selecting intervention strategies for behavioural change [36,37,38]. However, the COM-B model has yet to be applied to analyze barriers and enablers to attending LTFU care in the pediatric cancer context. The use of this model can help with increasing understanding on how various factors may interact to affect the attendance of LTFU care in survivors, guiding future interventions to address engagement issues.

The objective of this study is to identify the barriers and enablers to engaging in LTFU care of Canadian young adult pediatric cancer survivors, as reported by survivors and HCPs, guided by the COM-B model.

## 2. Materials and Methods

This study uses quantitative and qualitative methods. It is part of a larger project conducted through the University of Calgary, investigating young adult pediatric cancer survivors’ experience with engaging in cancer-specific LTFU care [39]. Only procedures relevant to this study will be reported. Ethical approval was granted through the Health Research Ethics Board of Alberta—Cancer Committee (HREBA.CC-20-0248).

### 2.1. Patient and Public Involvement

This patient-oriented project was developed in collaboration with our patient partners, who had longstanding relationships with the research team [39]. They were actively engaged in all aspects of the research process. Patient partners contributed to the study design and recruitment, co-facilitated focus groups and semi-structured interviews, participated in the analysis of the qualitative data, and were involved in knowledge dissemination (e.g., co-presenters at community events [39]).

### 2.2. Participants

Survivor eligibility criteria included (1) young adults aged 18–39 years, (2) diagnosed with cancer at <18 years of age, (3) >5 years post-diagnosis and/or >2 years post-treatment completion, and (4) living in Canada. HCP eligibility criteria included (1) HCPs (e.g., oncologists, general practitioners, advanced practice nurses, social workers) identified as delivering care to pediatric cancer survivors, (2) practicing for >5 years, and (3) living in Canada.

### 2.3. Recruitement

Survivor and HCP participant recruitment was performed through three main avenues to recruit participants from diverse sociodemographic backgrounds. First, both survivor and HCP participants were recruited via LTFU clinics across Canada and were informed about the study verbally via their LTFU care team and provided with an information package. Both groups then completed a consent-to-contact form and were contacted by the study team. Secondarily, both groups were recruited using social media strategies including X (formerly known as Twitter; X Corp, Bastrop, TX, USA), Facebook (Meta Platforms, Menlo Park, CA, USA), and Instagram (Meta Platforms, Menlo Park, CA, USA). Study graphics and information were shared on these platforms via patient advocacy groups (e.g., Childhood Cancer Survivor Canada). Finally, survivors were recruited via patient partners across Canada who were core members of the research team and distributed recruitment material within the study team’s respective networks.

### 2.4. Procedure

Survivor and HCP participants were emailed an online questionnaire link through Research Electronic Data Capture (REDCap, Nashville, TN, USA). Participants provided informed consent before beginning the survey and then completed a 45 min questionnaire covering demographic and clinical information, as well as barriers and enablers to attending long-term follow-up (LTFU) care. These survey questions were adapted from the APA cultural formulation interview framework which allowed participants to highlight how their cultural and social context may shape their engagement with LTFU care [40]. Participants who completed the survey and consented to follow-up were invited to take part in an audio-recorded semi-structured focus group or interview. Eligible participants provided consent prior to participation via a REDCap link. Focus groups with survivors were conducted virtually via Zoom and lasted 90 min, while semi-structured telephone interviews were facilitated with HCPs and lasted 20 min. Both groups received a CAD 25 gift card for their participation.

### 2.5. Measures

#### 2.5.1. Barriers and Enablers to Attending LTFU Care

**Survey.** Survivors and HCPs were asked about barriers and enablers via a multiple-choice survey (e.g., “From your view, has anything prevented/helped you/your patients from getting follow-up care?”). Barriers listed were “money”, “work commitment”, “family commitment”, “stigma or discrimination”, “language or background”, “transportation, “location of services”, “unfamiliar or distrust of healthcare team”, “other”, or “none at all”. Enablers listed were “support from family”, “support from friends”, “support from healthcare team”, “support from employer”, “money”, “familiarity/trust of healthcare team”, “location of services”, “flexibility of service delivery”, “other”, or “none at all”.

**Focus Groups and Interview Questions.** In survivor focus groups and HCP interviews, participants were asked about barriers and enablers to attending LTFU care (e.g., “What are some barriers/enablers to your follow-up care, in other words what things have been unhelpful/helpful”, “What would be some barriers/enablers, so things that can be either unhelpful/helpful, when you’ve delivered care to this population?”).

#### 2.5.2. Participant Characteristics

Demographic information collected from survivors and HCPs included age, sex, gender, ethnicity, and residence. Clinical information collected from survivors included diagnosis, age at diagnosis, years since treatment, and current health problems related to diagnosis or treatment. Professional information collected about HCPs included profession, training background, and length of time working with survivors.

### 2.6. Analysis

Quantitative data collected from the sociodemographic surveys and the enablers and barriers to LTFU care survey were managed and analyzed descriptively in IBM SPSS (Version 29). Qualitative data was analyzed using a two-phase approach of inductive and deductive analysis, an approach being increasingly used for behaviour change intervention development [41,42,43]. Qualitative data was audio-recorded, transcribed verbatim, and de-identified. Transcripts were analyzed using reflexive thematic analysis by a research team (HW, SH, BH, RD, KM) who were trained by SH with the help of RD who had previous experience in this analysis approach [44]. Each member of the team familiarized themselves with the transcripts and generated initial codes identifying major themes guided by the COM-B Model. The research team then compared their individual codes and created a consensus codebook to guide analysis. Next, each member of the research team analyzed the same transcript and then met as a group to ensure consistency of understanding of the codebook. After this, two members of the team individually analyzed each transcript, then met as a pair to compare coding and resolve any conflicts. Next, the research team met to review coding, resolve any ongoing conflicts, and extract major themes for the COM-B model. Then, HW used the COM-B model as a framework to converge qualitative and quantitative findings, which were both organized into the broad categories of *Capability*, *Opportunity*, and *Motivation* and then further into the sub-categories of *Physical* or *Psychological Capability*, *Physical* or *Social Opportunity*, and *Reflective* or *Automatic Motivation*. HW then identified the overall major themes mapped onto the COM-B model.

## 3. Results

### 3.1. Participant and Clinical Characteristics

#### 3.1.1. Survivors

**Survey**. One hundred and eight survivors completed the survey. The mean age of participants was 28.30 years (SD = 5.3 years). Most participants (70.4%, *n* = 76) indicated that their assigned sex at birth was female and identified as female. Most participants (78.7%, *n* = 85) identified as White/European. Participants reported an average age at cancer diagnosis of 9.71 years (SD = 5.34 years) and were on average 16.60 years (SD = 7.34) post-treatment.

**Focus Groups.** Twenty-two survivors completed the focus groups. The mean age of participants was 29.19 years (SD = 4.78). Most participants (95.5% *n* = 21) were assigned female at birth and identified as female. Most participants (86.4%, *n* = 19) identified as White/European. Participants reported an average age of cancer diagnosis at 10.59 years (SD = 5.45) and were on average 17.45 years (SD = 6.81) post-treatment. A summary of participant demographics and clinical characteristics is provided in Table 1.

#### 3.1.2. Healthcare Providers

**Survey**. Twenty HCPs completed the survey. The mean age of participants was 48.3 years (SD = 9.4 years). Overall, 80% (*n* = 16) of participants were assigned female at birth and identified as female, and 100% (*n* = 20) of participants identified as White/European. HCP roles included registered nurses (30%, *n* = 6), allied health professionals (30%, *n* = 6), oncology physicians (25%, *n* = 5), and nurse practitioners (15%, *n* = 3). Most participants had been providing care for survivors for 5–9 years (40%, *n* = 8) or for 1–4 years (30%, *n* = 6).

**Focus Groups**. Seven HCPs completed a semi-structured phone interview. The mean age of participants was 50.1 years (SD = 7.7 years). The majority (85.7%, *n* = 6) of participants’ assigned sex at birth was female and identified as female. Overall, 57.1% (*n* = 4) worked as allied health professionals and 42.9% (*n* = 3) as registered nurses. Most participants had been providing care for survivors for 5–9 years (42.9%, *n* = 3) or for 1–4 years (28.6%, *n* = 2). A summary of HCP demographics and clinical characteristics is provided in Table 1.

### 3.2. Perceived Barriers and Enablers to Engaging with Follow-Up Care

**Figure 1:**
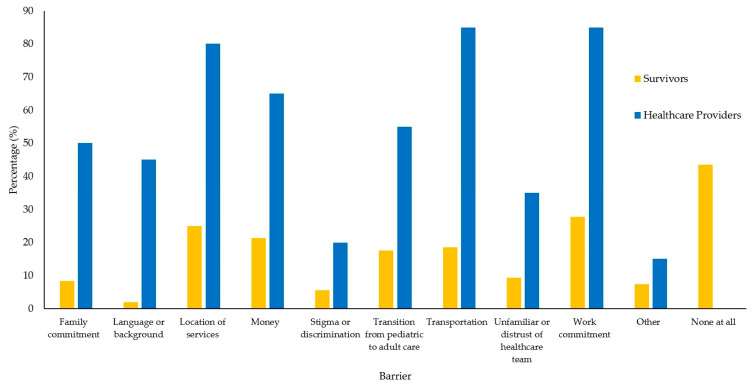
Percentage of survivors and healthcare providers that identified each factor as a barrier to attending long-term follow-up care.

**Figure 2:**
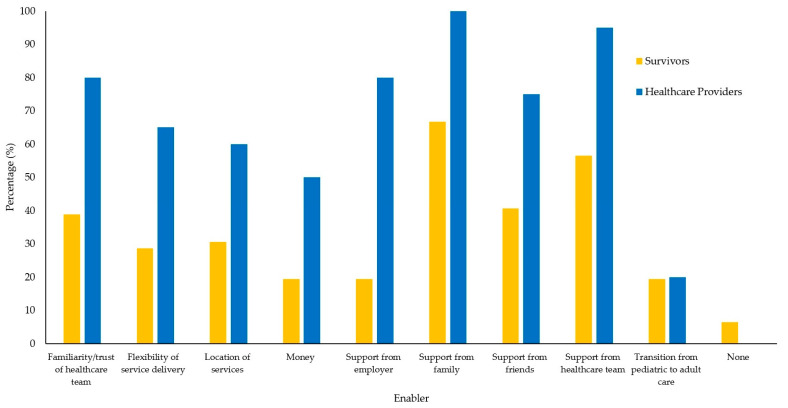
Percentage of survivors and healthcare providers that identified each factor as an enabler to attending long-term follow-up care.

**Figure 3:**
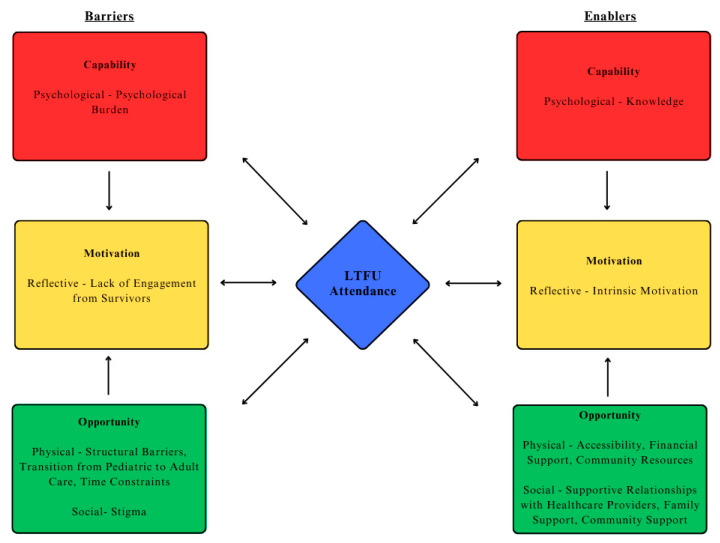
Barriers and enablers to long-term follow-up care attendance mapped onto the COM-B model.

Quantitative survey results identifying the proportion of participants endorsing factors as barriers and enablers are mapped in Figure 1 and Figure 2. A comprehensive summary of the quantitative and qualitative data mapped onto the COM-B model is provided in Table 2. The major themes are mapped onto the COM-B model in Figure 3.

#### 3.2.1. Capability: Psychological

**Barrier: Psychological Burden.** Survivors spoke about the psychological burden of attending LTFU care. They endorsed how attending LTFU provokes anxiety and worry, especially while waiting for test results or potentially receiving bad news.

*“I went to aftercare, I would say sporadically. And then just found it very anxiety provoking and miserable experience all around. And did not find any benefit in it.”—Survivor Participant ID 128*

One survivor highlighted how cancer had defined their life for so long and that attending LTFU prevented them from moving on with their life.

*“There came a point where I just was fed up with living in the cancer system, and, for better or worse, was willing to talk the risk of I don’t want to do this- I want to have a life.”—Survivor Participant ID 128*

**Enabler: Knowledge**. Survivors spoke about the importance of educating themselves about LTFU care. They endorsed how crucial it was to ask questions and clarify information about their care, as this knowledge helps them to advocate for their LTFU care and ensure they received the necessary treatment surveillance.

*“Going back to the enabling question, I think knowledge. The medical professionals being very open about my condition, what everything meant. Giving me that education to be able to understand what’s going on and not being scared to ask questions it allowed me to advocate for myself because I knew what I was talking about.”—Survivor Participant ID 144*

#### 3.2.2. Opportunity: Physical

**Barrier: Structural Barriers**. “Location of Services” was endorsed by 25% of survivors and 80% of HCPs as a barrier to LTFU care. “Transportation” was endorsed by 18.5% of survivors and 85% of HCPs as a barrier to LTFU care. Survivors and HCPs endorsed how survivors often need to travel large distances to attend LTFU care. This resulted in a large financial cost for survivors due to the cost of gas, toll fees, and accommodations. Likewise, “Money” was endorsed by 21.3% of survivors and 65% of HCPs as a barrier to LTFU care.

*“One of the biggest barriers is just geographic I think, and that ties into financial, because for some families that trip down to [city] in a location that’s very expensive to get down to and to stay at”—HCP Participant ID 2*

Survivors also endorsed the difficulty that arises with accessing LTFU care after moving provinces, due to the difference in how LTFU care is delivered across each province.

*“Physically moving to provinces is a huge barrier now, that I’ve learned, and encountering disparity in the way clinics in provinces may approach aftercare is a huge piece of that.”—Survivor Participant ID 77*

**Barrier: Transition from Pediatric to Adult Care.** “Transition from Pediatric to Adult Care” was endorsed by 17.6% of survivors and 55% of HCPs as a barrier to LTFU care. Survivors endorsed how they felt supported by the pediatric cancer healthcare system, but had to learn how to navigate the adult healthcare system on their own as a young adult. They highlighted their negative perception of the adult cancer care culture and the contrast to the pediatric environment. HCPs endorsed how the lack of structure and protocol in adult care, along with a lack of resources, makes it difficult for patients to engage with LTFU care during this period.

*“I find that when you’re in the pediatric world, there is a lot of people that do a lot of hand-holding and help you to navigate the world that you’re working with within ped. But then when you turn to be an adult, they kind of throw you to the wolves and expect you to figure it out.”—Survivor Participant ID*

**Barrier: Time Constraints**. “Family Commitment” was endorsed by 8.3% of survivors and 50% of HCPs, and “Work Commitment” was endorsed by 27.8% of survivors and 85% of HCPs, as barriers to LTFU care. Survivors explained how they have busy lives and finding time to attend LTFU care appointments, especially due to the limited availability of these appointments, can be very difficult. Some survivors highlighted how they have to take a full day off work to attend their LTFU care appointments.

*“For me, the barriers in just getting, it was for follow-up appointments, was just the time.—Survivor ID 59*

**Enabler: Accessibility.** “Location of Services” was endorsed by 30.6% of survivors and 60% of HCPs, and “Flexibility of Service Delivery” was endorsed by 28.7% of survivors and 65% of HCPs, as enablers to LTFU care. Survivors and HCPs endorsed the benefit of virtual appointments and the formats’ ability to accommodate a survivor’s busy life and reach survivors in a larger geographical area.

*“Because we’re a provincial program, one of the interesting things about doing everything virtually is it sort of flattens the access more. So the ability to connect with people is kind of equalized, whether you live in [location] and [location] or you live in [location] it doesn’t matter, I can see you by phone or by Zoom either place.”—HCP ID 80*

Furthermore, survivors and HCPs endorsed the usefulness of email reminders and pre-scheduling appointments to ensure survivors attended their appointments.

*“I think the biggest enabler for actually having these appointments for me is that they’re scheduled for me and then they’re put in my like hospital calendar app.—Survivor ID 44*

**Enabler: Financial Supports.** “Money” was endorsed by 19.4% of survivors and by 50% of HCPs as an enabler to LTFU care. One survivor spoke about how specific forms of financial support (e.g., gas cards) helped to eliminate the financial barriers to attending LTFU care.

*“We would always have to book a hotel room and things like that, and the social worker was great in helping us. So it was kind of they had support, like if they had a gas card they can help with that.”—Survivor ID 85*

**Enabler: Community Resources.** HCPs frequently endorsed the importance of community organizations and their role in ensuring survivors received comprehensive LTFU care. These organizations provided counselling, mental health supports, tutoring, and recreational activities to survivors, which often filled in the gaps of what the healthcare system could provide to survivors.

*“Like I said, huge is mental health supports. I mean, we do have the [organization], we’re very lucky to have the [organization], that is an external organisation that provides some counselling.”—HCP ID 2*

#### 3.2.3. Opportunity: Social

**Enabler: Supportive Relationships with Healthcare Providers.** “Familiarity/trust of Healthcare Team” was endorsed by 38.9% of survivors and 80% of HCPs as an enabler to LTFU care. Survivors spoke about how their long-term relationships with their healthcare team were one of the major reasons they attended LTFU care. Survivors spoke about how they especially appreciated seeing the same HCPs at each appointment.

*“Seeing those familiar faces, was definitely one of the reasons that when I did go, was one of the positives.”—Survivor ID 128*

“Support from Healthcare Team” was endorsed by 56.5% of survivors and 95% of HCPs as an enabler to LTFU care. Survivors spoke about how their trust in their HCPs enabled them to feel confident with the quality of their LTFU care. They highlighted how the connection that they built with their HCPs helped them feel confident with asking questions and expressing their concerns in LTFU care.

*“I have a great relationship with my care team, it makes the world of difference, because you do feel safe and you don’t feel silly in going for little things.”—Survivor ID 85*

**Enabler: Family Support.** “Support from Family” was endorsed by 66.7% of survivors and 100% of HCPs as an enabler to LTFU care. Survivors spoke about the assistance their family provided when getting to and from their LTFU appointments. They also described how their families’ strong belief in the importance of long-term follow-up (LTFU) care influenced their own recognition of its value.

*“Ultimately as a young person, your family is getting you to those appointments and they’re also helping you see the value of them, too…at least from my experience, part of why I also kept going to aftercare was because it was important to my mom and my family.”—Survivor ID 77*

**Enabler: Community Support.** “Support from Employer” was endorsed by 19.4% of survivors and 80% of HCPs, and “Support from Friends” was endorsed by 40.7% of survivors and 75% of HCPs, as enablers to LTFU care. Survivors endorsed the importance of building relationships with fellow survivors, as this provided a chance to share their feelings with people who have been through similar experiences. Some survivors endorsed how they felt they were providing a sense of hope to current cancer patients when attending their LTFU appointments.

*“I also feel like sometimes when you go and, yes, you are surrounded by children who are going through it, it takes you back but you’re also “hey, these people, these kids, they look at me and they’re ‘hey, I can beat this, right?” So having you go there it’s almost like you’re giving them a sense of hope.”—Survivor ID 87*

#### 3.2.4. Motivation: Reflective

**Barrier: Lack of engagement from survivors.** HCPs spoke about how the perceived lack of engagement from young survivors often acts as a barrier. They explained how, as children, many survivors had help from their family to book appointments, but as adults, they find it difficult to take control of their healthcare. HCPs also endorsed how some survivors do not want to attend LTFU appointments or do not show up to these appointments.

*“I would say another barrier because it’s that transition from care as a child, or a young person, into the adult world we really take the perspective of, you’re the adult now so it’s your care, you need to be engaged.”—HCP Participant ID 80*

**Enabler: Intrinsic Motivation**. Survivors spoke about how they have a strong belief in the importance of attending LTFU care. They endorsed how their personal desire to keep themselves healthy encouraged them to attend their LTFU appointments. They also spoke about their confidence in their LTFU care, and how regularly attending appointments ensured that any issues that arose would be caught and treated.

*“There’s no incentive for me to go, it’s just other than keeping myself healthy.”—Survivor Participant ID 6*

Finally, 43.5% of survivors, but no HCPs, reported that survivors experience no barriers to LTFU care.

## 4. Discussion

Many young adult survivors of pediatric cancer do not attend LTFU care, indicating a need to better understand the barriers and enablers that survivors face, from the perspectives of survivors as well as healthcare providers. Accordingly, we conducted a study using the COM-B model as a theoretical base to identify barriers and enablers that survivors of pediatric cancer experience when engaging with LTFU care.

Our findings aligned with the previous literature identifying structural barriers (e.g., cost, distance) as significant barriers that influence survivors’ *physical opportunities* to attend LTFU care [3,7,8,9,11,12,13,14,16,17,18,19,20,21]. Notably, our study highlights that these structural barriers are often influenced by each other, reinforcing emerging findings that the financial challenges to attending LTFU care in Canada often arise from the need for survivors to travel long distances to attend this care [20]. This is especially relevant in Canada, where the cost of LTFU is covered by universal healthcare, but where LTFU clinics provide care for large geographical regions. Despite the absence of direct care costs, this indicates where a larger proportion of financial costs may occur (i.e., gas cost, hotels, food, transport) when attending LTFU care in Canada. These findings may be consistent with the experiences of survivors in other countries with universal healthcare coverage, indicating places for interventions to target. However, there must be country-specific considerations that are factored into the development of such strategies.

One prominent enabler that arose from our study was the importance of supportive relationships with survivors and their healthcare team to increase survivors’ *social opportunity* to engage with LTFU care. This adds to the literature about the importance of building strong relationships between survivors and their HCPs [3]. Physician–patient relationships have been shown to affect a patient’s adherence in healthcare [45]. However, creating a trusting relationship between a survivor and an HCP can be difficult. In our study, survivors valued HCPs taking the time to ask about and note details of their personal lives, which helped strengthen the patient–provider relationship and supported continued LTFU attendance. This practice of asking about and recording notes about patients’ personal lives is part of the “preparing with intention” method, a method suggested in the literature that can help physicians build meaningful connections with patients [46].

*Community support* was identified as an enabler to increasing *social opportunity* to engage with LTFU care. The importance of community cancer organizations has been echoed in previous studies during the transition from pediatric to young adult care for pediatric cancer survivors [19]. Our study indicates the continued importance of a relationship between community organizations and HCPs across the continuum of LTFU care. The issue arises that it can be difficult for HCPs who provide LTFU care to residents of a large area to stay up to date with community resources offered for survivors [47]. This could result in survivors not accessing community resources that may provide them with accessible LTFU care. HCPs can enhance their awareness of community-based resources through interdisciplinary collaboration, particularly with social work colleagues who often possess current knowledge of local services. Additional strategies include soliciting feedback from patients regarding supports they have found beneficial and proactively engaging with community organizations to stay informed about available programs. These findings suggest the importance of including community resources in future interventions to ensure survivors and HCPs are aware of all potential resources at their disposal.

Our work was unique in applying the COM-B model to examine barriers and enablers to LTFU care for young adult survivors of pediatric cancer. Building on existing work, our application of the COM-B model facilitated a greater understanding of how survivors’ experience with attending LTFU care was most affected by their *opportunity* to attend LTFU care. Survivors highlighted how major barriers (structural barriers, transitioning from pediatric to adult care, and time constraints) affected their *physical opportunity* to engage with LTFU care, while enablers (accessibility, financial support, supportive relationships with healthcare providers and family, and community support) enhanced their *physical and social opportunities* to participate in LTFU. This indicates key areas that would be beneficial to address future interventions to improve LTFU care engagement. Furthermore, the COM-B model enhanced our understanding of how certain barriers and enablers may interact to influence attendance in LTFU care. For example, the COM-B model recognizes how an individual’s *motivation* to participate in a target behaviour is often influenced by their *capability* and *opportunity* to participate in that behaviour [35]. Consistent with this, survivors in our study spoke about how their intrinsic belief in the importance of LTFU care (*motivation*) was an enabler to attending LTFU care (*behaviour*). Survivors spoke about the importance of family support and how they learned the importance of LTFU care from their parents (*opportunity*). Thus, young adult survivors’ *opportunity* to learn from their parents about LTFU care importance resulted in more intrinsic *motivation* to engage in LTFU care (*behaviour*) (Figure 3).

Another strength of this study was the use of both quantitative and qualitative methods which allows for a greater understanding of the issues that address survivors [48]. The qualitative data provided a richer description of why participants reported certain barriers and enablers to care, and allowed us to interpret the data using a different lens. Meanwhile, the larger quantitative sample allows for generalization of our findings to a larger population [49]. Our sample is also geographically representative, providing another strength to our study. Research has shown that adult survivors of pediatric cancer who live rurally may experience more barriers to LTFU care than urban counterparts [20] and are more likely to receive sub-optimal LTFU care compared to their urban counterparts [50]. McKenzie et al. reported that approximately 20% of Canadian survivors reside in rural areas, a proportion closely mirrored in our sample, in which 20.4% of survivor participants identified as living rurally or remotely [51]. This alignment suggests that our sample is geographically representative and supports the generalizability of our findings to both rural and urban survivor populations.

Our quantitative findings revealed that HCP consistently perceived more barriers to accessing LTFU care than survivors themselves, with a notable number of survivors highlighting that they experienced no barriers at all to engaging with care. This discrepancy could be attributed to our recruitment methods utilized in this study, with the majority of our participants being recruited from LTFU clinics. These participants are already accessing LTFU care, and may not perceive as many barriers as someone who is not currently accessing such services. Previous intervention design has often lacked direct survivor involvement [39]. However, our study shows that the experiences and perceptions of survivors may not align with the perceived experiences by HCPs, reinforcing the importance of survivor involvement in intervention design.

### Limitations and Future Directions

We recognize a potential bias in that our study lacked representation from diverse backgrounds, as the majority of our sample identified as white and female despite intentional efforts to recruit participants from diverse backgrounds. It is well known that female-identifying survivors outnumber male-identifying participants in LTFU, which was reflected in our study, with 70.4% of survivors identifying as female, highlighting a large group of survivors not attending LTFU care who are not encompassed in our study [52]. In addition, non-white survivors are less likely to engage with LTFU care than their white counterparts [52] and face additional barriers and stigma when accessing LTFU care [53]. This crucial concept was unfortunately not captured in our study, even when providing language and stigma as prompts in the quantitative survey. This highlights important barriers not captured in our data and emphasizes the need to collaborate with survivors to develop culturally sensitive interventions that enhance engagement across diverse populations. These limitations underscore the need for future research to develop and implement intentional recruitment strategies that ensure greater participant diversity, with particular attention to individuals not currently engaged in LTFU care.

Furthermore, findings from this study may help to inform future interventions that work to improve engagement in LTFU care among young adult survivors. Emerging evidence suggests that mobile health (mHealth) interventions hold promise as a potential strategy to enhance such engagement in this population, offering further potential areas for targeted focus [39].

## 5. Conclusions

This study used the COM-B model to identify barriers and enablers of LTFU care for Canadian survivors of pediatric cancer, as discerned by survivors and HCPs. Survivors highlighted how their *opportunity* to engage in LTFU largely influences their ability to engage with LTFU care. Structural barriers, transitioning from pediatric to adult care, and time constraints were highlighted as barriers that prevent survivors’ *physical opportunity* to engage in LTFU care. Accessibility, financial support, supportive relationships with healthcare providers and family, and community support were highlighted as enablers that assist survivors with the *physical and social opportunity* to engage in LTFU care.

## Figures and Tables

**Table 1:** Demographic and clinical characteristics of survivors who participated in the survey (*n* = 108) and focus groups (*n* = 22) and demographic and professional characteristics of healthcare providers who participated in the survey (*n* = 20) and interviews (*n* = 7).

Survivors of Pediatric Cancer—Demographic and Clinical Characteristics	Survey Participants *n* = 108		Focus Group Participants *n* = 22	
	*n (%)*	*M (SD)*	*n (%)*	*M (SD)*
**Current age (in years)**	105	28.30 (5.27)	21	29.19 (4.78)
**Sex**				
Male	32 (29.6)		1 (4.5)	
Female	76 (70.4)		21 (95.5)	
**Gender**				
Male	31 (28.7)		1 (4.5)	
Female	74 (68.5)		21 (95.5)	
Gender Fluid, Non-binary or Two-Spirit	3 (2.8)			
**Ethnicity**				
Aboriginal/First Nations/Inuit/Métis	3 (2.8)		2 (9.1)	
Black/African/Caribbean	3 (2.8)		1 (4.5)	
East Asian	14 (13.0)		2 (9.1)	
Latin American	1 (0.9)			
Middle Eastern	3 (2.8)			
South Asian	5 (4.6)			
White/European	85 (78.7)		19 (86.4)	
Other	2 (1.9)		2 (9.1)	
**Province of Residence**				
Alberta	38 (35.2)		10 (45.5)	
British Columbia	37 (34.3)			
New Brunswick	2 (1.9)		1 (4.5)	
Nova Scotia	5 (4.6)		4 (18.2)	
Ontario	19 (17.6)		5 (22.7)	
Quebec	2 (1.9)		1 (4.5)	
Saskatchewan	1 (0.9)		1 (4.5)	
Yukon	1 (0.9)			
No Response	3 (2.8)			
**Geographic Region**				
Rural	20 (18.5)		4 (18.2)	
Urban	83 (76.9)		18 (81.8)	
Remote	2 (1.9)			
No Response	3 (2.8)			
**Age at Diagnosis** (in years)	108	9.71 (5.34)		10.59 (5.45)
**Years post-treatment**	108	16.60 (7.43)		17.45 (6.81)
**Cancer Diagnosis**				
Leukemia (e.g., ALL, AML)	36 (33.3)			11 (50)
Lymphoma (e.g., Hodgkin’s, non-Hodgkin’s)	22 (20.4)			6 (27.3)
Solid Tumour (e.g., Wilms tumour,	23 (21.3)			3 (13.6)
osteosarcoma)				
Brain Tumour (e.g., Medulloblastoma)	12 (11.1)			
Other	15 (13.9)			2 (9.1)
**Recruitment**				
Hospital/Clinic	50 (46.3)			2 (9.1)
Twitter/Facebook/Instagram/Internet	14 (13.0)			6 (5.6)
Colleague	4 (3.7)			
Family/Friend	7 (6.5)			2 (9.1)
Community Organization	9 (8.3)			1 (4.5)
No Response	24 (22.2)			11 (50.0)
**Healthcare Providers—Demographic and Professional Characteristics**	Survey Participants (*n* = 20)		Interview Participants (*n* = 7)	
**Current Age** (in years)	20	48.3 (9.41)	7	50.14 (7.73)
**Sex**				
Male	4 (20.0)		1 (14.3)	
Female	16 (80.0)		6 (85.7)	
**Gender**				
Male	4 (20.0)		1 (14.3)	
Female	16 (80.0)		6 (85.7)	
**Ethnicity**				
White/European	20 (100.0)		7 (100.0)	
**Profession**				
Registered Nurse	6 (30.0)		3 (42.9)	
Allied Health Professional	6 (30.0)		4 (57.1)	
Nurse Practitioner	3 (15.0)			
Physician-Oncologist	5 (25.0)			
**Years Working with Survivors of Pediatric Cancer**				
1–4 years	6 (30.0)		2 (28.6)	
5–9 years	8 (40.0)		3 (42.9)	
10–14 years	1 (5.0)		1 (14.3)	
15 or more years	5 (25.0)		1 (14.3)	
**Province of Residence**				
Alberta	6 (30.0)		2 (28.6)	
British Columbia	5 (25.0)		3 (42.9)	
Manitoba	2 (10.0)		1 (14.3)	
Nova Scotia	2 (10.0)			
Ontario	4 (20.0)		1 (14.3)	
Prefer Not to Answer	1 (5.0)			
**Geographic Region**				
Urban	18 (90.0)		7 (100.0)	
Rural	2 (10.0)			

**Table 2:** Survey responses and qualitative themes mapped to the COM-B model for survivors and healthcare providers.

COM-B Model	Barriers			Enablers			
	Quantitative (Survey)		Qualitative (Focus Group/Interview)	Quantitative (Survey)			Qualitative (Discussion Group/Interview)
	Survivors	HCPs		Survivors	HCPs		
**Capability—Individuals’ physical and psychological capacity to engage in an activity**							
***Physical*** (Individuals’ physical capacity (skills, ability) to engage in an activity)			**HCP capacity (HCP):** Limited capacity of HCP to provide high-quality LTFU care.				
***Psychological*** (Individuals’ capability (memory, knowledge, confidence) to engage in the thought processes of an activity	**Language or background**		**Psychological burden (Survivors)**: Emotional impact of attending LTFU care.				**Knowledge (Survivors):** Importance of survivors educating themselves on LTFU to advocate for their LTFU care.
	*n* = 2 (1.9%)	*n* = 9 (45%)					
**Opportunity—External factors that enable or prevent a behaviour**							
***Physical*** (Environmental factors that influence a behaviour)	**Family commitment**		**Structural barriers (Survivors and HCP):** Distance, cost, and location of LTFU care. **Time (Survivors):** Finding time in their schedules to attend LTFU care. **Transition to adult care (Survivors and HCP):** Lack of support in the adult system, negative culture, lack of specific resources.	**Flexibility of service delivery**			**Accessibility (Survivors and HCP):** Format of care, reminders. **Financial supports (Survivors):** Financial supports (e.g., gas cards). **Community resources (HCPs):** Community organizations providing extra supports (e.g., counselling, recreational activities). **Increase in system funding (HCPs):** Provide more mental and physical support.
	*n* = 9 (8.3%)	*n* = 10 (50%)		*n* = 31 (28.7%)		*n* = 13 (65%)	
	**Location of services**			**Location of services**			
	*n* = 27 (25%)	*n* = 16 (80%)		*n* = 33 (30.6%)		*n* = 12 (60%)	
	**Money**			**Money**			
	*n* = 23 (21.3%)	*n* = 13 (65%)		*n* = 21 (19.4%)		*n* = 10 (50%)	
	**Transition from pediatric to adult care**			**Transition from pediatric to adult care**			
	*n* = 19 (17.6%)	*n* = 11 (55%)		*n* = 21 (19.4%)		*n* = 4 (20%)	
	**Transportation**						
	*n* = 20 (18.5%)	*n* = 17 (85%)					
	**Work commitment**						
	*n* = 30 (27.8%)	*n* = 17 (85%)					
***Social*** (Cultural norms, interpersonal influences that influence a behaviour)	**Stigma or discrimination**		**Stigma (Survivors):** Issue accessing tests	**Familiarity/trust of healthcare team**			**Supportive relationships with healthcare providers (Survivors):** Building trusting relationships with providers. **Family support (Survivors):** Taught the importance of attending LTFU care by family, assistance with travelling to appointments. **Community support (Survivors):** Creating a community with other survivors.
	*n* = 6 (5.6%)	*n* = 4 (20%)		*n* = 42 (38.9%)		*n* = 16 (80%)	
				**Support from employer**			
				*n* = 21 (19.4%)		*n* = 16 (80%)	
				**Support from family**			
				*n* = 72 (66.7%)		*n* = 20 (100%)	
				**Support from friends**			
				*n* = 44 (40.7%)		*n* = 15 (75%)	
				**Support from healthcare team**			
				*n* = 61 (56.5%)		*n* = 19 (95%)	
**Motivation—Brain processes that energize and direct behaviour**							
***Reflective*** (Involves evaluations and plans about a behaviour)			**Lack of engagement from survivors (HCP):** Perceived lack of interest from survivors in their LTFU care.				**Intrinsic motivation (Survivors):** Internal belief that LTFU care and their health is important.
***Autonomic*** (Emotions and impulses that arise from associative learning and/or innate disposition)	**Unfamiliar of distrust of healthcare team**						
	*n =* 10 (9.3%)	*n* = 7 (35%)					

## Data Availability

Data generated or analyzed during this study are available from the corresponding author on reasonable request.
